# Case Report: A case of reversible tracheal diameter Mounier-Kuhn syndrome and literature review

**DOI:** 10.3389/fmed.2025.1544843

**Published:** 2025-04-28

**Authors:** Lu-xia Kong, Zhen-hua Li, Ji-xiang Ni

**Affiliations:** The Central Hospital of Wuhan, Tongji Medical College, Huazhong University of Science and Technology, Wuhan, China

**Keywords:** tracheobronchomegaly, Mounier-Kuhn syndrome, reversible, tracheal diameter, tracheomegaly

## Abstract

Mounier-Kuhn syndrome (MKS), also known as tracheobronchomegaly (TBM) or tracheomegaly, is an extremely rare and chronic airway disease characterized by significant dilation of the trachea and central bronchi. Currently, there is a paucity of epidemiological studies on MKS, with the majority of data derived from case reports, resulting in limited understanding of this disease among clinicians. We encountered an 81-year-old male MKS patient whose diagnosis was delayed, and subsequently experienced a significant decrease in tracheal diameter post-diagnosis, marking the first documented case of reversible tracheal diameter in MKS patients. Concurrently, we conducted a review of 76 cases published within the past decade to procure comprehensive insights into the clinical characteristics of MKS patients. Based on our findings, we draw two key conclusions. First, at present, clinicians and radiologists have insufficient understanding of this disease, which often leads to missed diagnosis, misdiagnosis or delayed diagnosis. Second, the tracheal diameter of MKS has the potential to be reversible with appropriate treatment. This is the first case where the tracheal diameter has been found to be reversible.

## Introduction

Mounier-Kuhn syndrome (MKS), also known as tracheobronchomegaly (TBM) or tracheomegaly, is an extremely rare and chronic airway disease characterized by significant dilation of the trachea and central bronchi. The condition may be hereditary or acquired, and is associated with the atrophy of the airway elastic fibers, thinning of smooth muscle, and diverticulum formation between the cartilage rings.

MKS, a highly misdiagnosed condition, was first identified by Czyhlarz in an autopsy in 1897, but it was not until 1932 that Mounier-Kuhn proposed a link between airway dilation and recurrent respiratory infections (1). To date, approximately 400 cases have been described (1), with a prevalence between 0.4 and 1.6% (2).

MKS is a rare clinical-radiological entity that can be diagnosed radiographically through chest radiographs, computed tomography (CT) scans, endoscopically, or through bronchoscopy or tracheoscopy. Furthermore, during this bronchoscopy, punch biopsies can be taken and may show a loss of elastic fibers, atrophy of longitudinal muscles, and muscularis mucosae thinning (3).

For patients with MKS, discharge of secretions is affected due to defective clearance and ineffective cough mechanisms. As a result, recurrent lung infections and bronchiectasis often occur (4).

We report on a patient with MKS who presented to our hospital in 2023 with reversible airway changes. Given the rarity of the disease, it was not possible to collect enough cases in the clinic, so we decided to retrospectively analyze previously published cases to further explore the characteristics of the disease. We performed a literature search using the keywords ‘Mounier-Kuhn Syndrome,’ ‘Tracheobronchomegaly,’ ‘tracheomegaly’ or ‘Bronchomegaly’ to identify relevant case reports in the database (primarily PubMed) in the past decade by 16 October 2024. Only cases involving patients aged 18 years or older were included. Non-English articles and those lacking clear measurements of tracheal or main bronchial diameter were excluded. A total of 76 MKS cases were collected and analyzed.

## Case description

**Figure 1:**
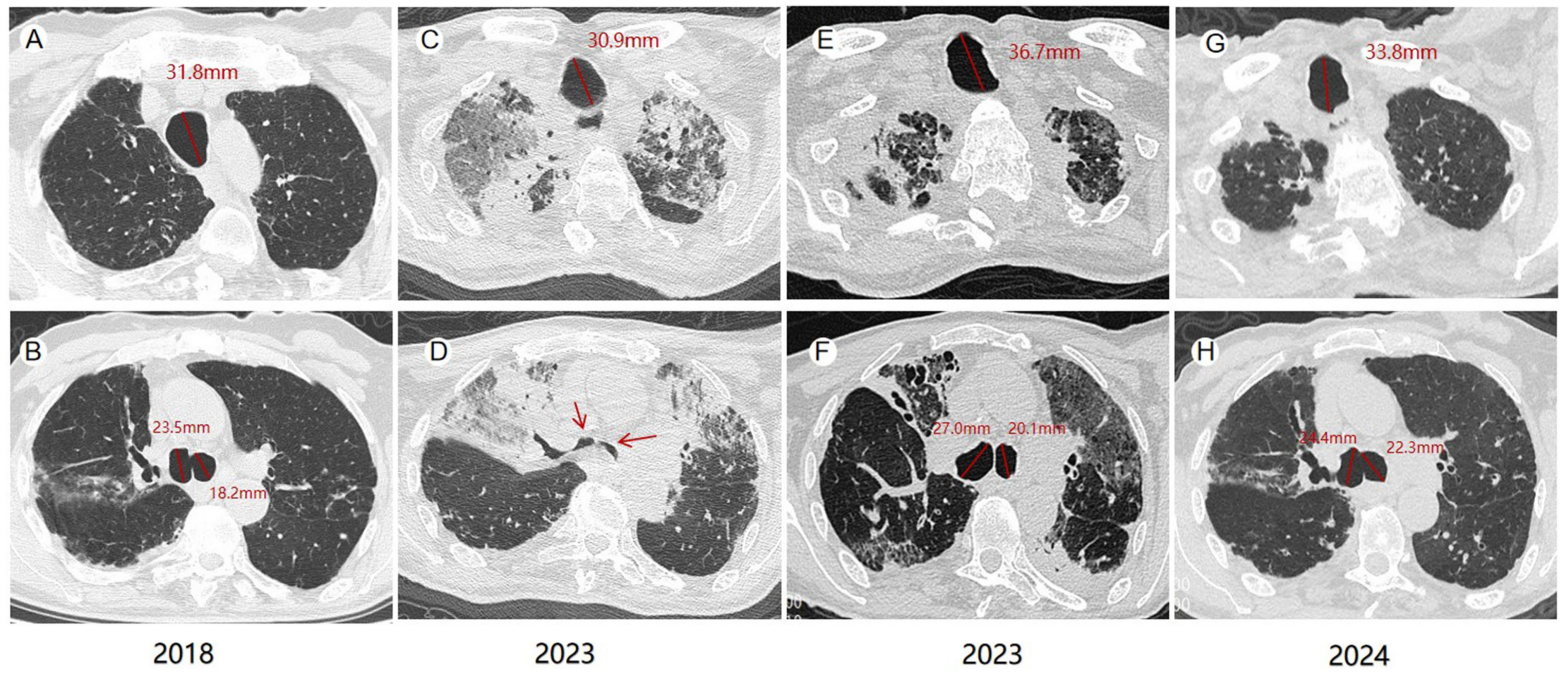
Computed tomography (CT) changes of the patient. In 2018, the maximum diameter of the patient’s trachea, right and left main bronchi were 31.8 mm, 23.5 mm and 18.2 mm **(A,B)**, respectively. Collapse of the tracheal walls during exhalation was seen on CT at admission in 2023 **(C,D)**. The maximum diameters of the trachea, right and left, main bronchi in 2023 were 36.7 mm, 27.0 mm and 20.1 mm, respectively **(E,F)**. Chest CT in 2024 showed that the maximum diameters of trachea, right and left main bronchi were 33.8 mm, 24.4 mm and 22.3 mm, respectively **(G,H)**.

On January 3, 2023, an 81-year-old male was admitted with dyspnea and decreased appetite for 1 week. The patient, a Chinese national, had pulmonary tuberculosis, treated surgically in 1964. Since 2018, the patient has been hospitalized in our hospital for several times due to acute exacerbation of chronic obstructive pulmonary disease, bronchiectasis with infection, and recurrent lung infections. The patient was conscious when he was admitted, and his oxygen saturation was 92% without oxygen. He had a thick breathing sound in both lungs, and wet crackles could be heard in the lower right lung. Blood routine examination showed white blood cells 11.76*10^9/L (3.5 ~ 9.5*10^9/L), neutrophil count 82.10% (40 ~ 75%), C-reactive protein 7.07 mg/dL (0 ~ 6 mg/L), interleukin 6 22.52 pg/mL (<7 pg/mL), procalcitonin 1.30 ng/mL (0 ~ 0.1 ng/mL). And novel coronavirus nucleic acid was positive. As shown in Figure 1, the patient’s chest CT showed double lung infection, bronchiectasis, and significant tracheobroncho enlargement. He was given symptomatic treatment such as high-flow oxygen inhalation, anti-virus, anti-infection, asthmatic relief, phlegm reduction and nutritional support. Two weeks later, the symptoms improved and the patient was discharged from the hospital. The chest CT examination 1 year later showed that the trachea and the right main bronchus were less enlarged than before.

## Discussion

MKS is a rare condition that is frequently overlooked due to its atypical symptoms and limited awareness among clinicians. In the case we present, the patient exhibited an enlarged trachea in 2018 but was not diagnosed until 2023. To enhance clinicians’ understanding of the condition, we conducted a review of cases over the past decade. In our study, 76 cases were included, and seriously, the average time from symptom onset to diagnosis was 14.24 ± 11.17 years, as detailed in Table 1 (see the Supplementary Table for the list of cases included).

**Table 1:** Characteristics of the eligible cases for analysis.

Variable	Statistics
Age (years), Mean ± SD	57.22 ± 17.43
Oldest	94
Youngest	21
Age distribution, n/total n (%)	
20–39 years	13/77 (17.88)
40–59 years	26/77 (33.77)
>60 years	38/77 (49.35)
Sex, n/total n (%)	
Male	64/77 (83.12)
Female	13/77 (16.88)
Smokers, n/total n (%)	16/77 (20.78)
Average tracheal diameter (mm), Mean ± SD	36.31 ± 7.95
Largest	62.6
Average bronchial diameter (mm), Mean ± SD	
Right bronchus	23.71 ± 5.41
Left bronchus	22.27 ± 5.13
Tracheal diverticulosis, n/total n (%)	39/77 (50.65)
Bronchiectasis, n/total n (%)	49/77 (63.64)
Tracheobronchial dyskinesia, n/total n (%)	16/77 (20.78)
Recurrent respiratory infections, n/total n (%)	50/77 (64.94)
Comorbidities, n/total n (%)	
PTB	3/77 (3.90)
COPD	12/77 (15.58)
IPF	3/77 (3.90)
Tumor	1/77 (1.30)
Pulmonary embolism	1/77 (1.30)
Three types of MKS, n/total n (%)	
Type 1	37/77 (48.05)
Type 2	18/77 (23.38)
Type 3	22/77 (28.57)
Lung function test, n/total n (%)	
Normal	9/40 (22.50)
Obstructive	21/40 (52.50)
Restrictive	8/40 (20.00)
Mixed	2/40 (5.00)
Symptoms, n/total n (%)	
Fever	16/77 (20.78)
Dry cough	16/77 (20.78)
Productive cough	44/77 (57.14)
Hemoptysis	15/77 (19.48)
Dyspnea	47/77 (61.04)
Chest pain	11/77 (14.29)
Weight loss	4/77 (5.19)
Anorexia	1/77 (1.30)
Pharyngeal discomfort	3/77 (3.90)
Respiratory failure	12/77 (15.58)
Dysphagia	1/77 (1.30)
Club-finger	6/77 (7.79)
Asymptomatic	2/77 (2.60)
Changes upon auscultation (wheezes, rhonchi, crepitation)	29/77 (37.66)
Unilateral	3/77 (3.90)
Bilateral	26/77 (33.77)

n, number of patients; SD, standard deviation; PTB, pulmonary tuberculosis; COPD, chronic obstructive pulmonary disease; IPF, idiopathic pulmonary fibrosis; n/total n denotes the available number/total number because of some missing data.

Consistent with previous findings, MKS predominantly affected males (5), with males accounting for 83.12% (64/77) of cases. In fact, in 1984, conventional chest X-ray showed that the size of the trachea was only related to gender, not weight or height (6). MKS is typically diagnosed in the 5th and 6th decades of life (4). The older the age group, the higher the incidence, patients >60 years old accounted for 49.35% (38/77). The oldest age was 94 years old. Smoking and exposure to air pollutants are significant risk factors (7). A history of smoking was reported in 16 cases (20.78%).

MKS can be diagnosed when chest CT shows (female/male) trachea transverse diameter > 21/25 mm, sagittal diameter > 23/27 mm, and when the trachea transverse diameter of the right and left main bronchi are >19.8/21.1 mm and > 17.4/18.4 mm, respectively (8). Krustins et al. reported an average tracheal diameter of 36 mm (range, 25–65 mm) in MKS patients (9). Our findings are consistent with this, as the average tracheal diameter in our cohort of 77 MKS cases was 36.31 ± 7.95 mm, with a maximum diameter of 62.6 mm. The average diameters of the right and left main bronchi were 23.71 ± 5.41 mm and 22.27 ± 5.13 mm, respectively. To date, few MKS cases with long-term follow-up have been reported, and in these cases, the tracheal diameter tends to increase progressively. In a single patient diagnosed with MKS, the tracheal diameter increased from 35 mm to 42 mm, the left bronchial diameter increased from 20 mm to 25 mm, and the right bronchial diameter increased from 18 mm to 23 mm over a seven-year follow-up period (10). Our patient represents the first documented case of reversible tracheal enlargement in MKS. From 2023 to 2024, the diameter of his trachea decreased from 36.7 mm to 33.8 mm, and the diameter of his right main bronchus decreased from 27.0 mm to 24.4 mm.

In addition, MKS is also diagnosed by the cross-sectional area of the trachea, which exceeds 371 mm^2^ in men and 299 mm^2^ in women (8). Interestingly, from a physiological perspective, airflow is inversely proportional to the fourth power of the radius. If the tracheal radius is increased by 2 times, the flow rate should be reduced by 16 times. The decrease in airflow rate leads to increased difficulty in clearing the airway, which leads to obstructions in removing secretions (11).

Bronchoscopy remains a strong diagnostic tool that can detect the dilation in the trachea and main bronchi during inspiration; their constriction, and even collapse, during expiration and coughing. This tracheobronchial dyskinesia may also manifest on CT in our patients, with 20.78% (16/77) exhibiting this finding. Biopsy of the trachea can show atrophy of elastic fibers and smooth muscle tissue or complete absence of cartilaginous elements (12).

Although pulmonary function testing is not considered diagnostic, some authors propose that it can assist in determining supportive care (3). Enlarged airways affect spirometry results due to weakness in tracheobronchial walls and hypotonia of myoelastic elements. These pathological changes lead to dynamic airway compression (manifested as expiratory collapse during forced exhalation) and dynamic restriction. Among the 40 patients who underwent pulmonary function testing, obstructive patterns were observed in 21 cases (52.5%), restrictive patterns in 8 (20%), mixed patterns in 2 (5%), with normal results in 9 patients (22.5%).

In addition to increased tracheal diameter, another common radiographic manifestation of MKS is airway diverticula. Diverticula were more frequently observed in the posterolateral wall, at the junction of the extrathoracic and intrathoracic parts of the trachea, which may be attributed to anatomical defects at this level or increased intracavitary pressure secondary to chronic cough (4, 8). Diverticula were present in 39 (50.65%) of 77 patients, and MKS could be classified into three types according to the presence or absence of diverticula and the anatomical distribution of diverticula. Type 1 is characterized by mild diffuse dilation of the trachea and main bronchi, while Type 2 exhibits abnormal and varying widenings accompanied by diverticula in the major airways. In Type 3, diverticula and sacculations accompany widening in the main airways extending to the distal bronchi (13). Among the 77 patients, type 1, type 2 and type 3 patients accounted for 48.05% (37/77), 23.38% (18/77) and 28.57% (22/77), respectively. Additionally, the tracheal diverticulum may serve as a reservoir for secretions, exacerbating the illness in MKS patients and increasing their susceptibility to recurrent respiratory infections (64.94%) and bronchiectasis (63.64%) (14).

The clinical presentations of MKS patients were diverse, including asymptomatic cases (2.60%). Typical symptoms resembling lung infection included: fever (20.78%), dry cough (20.78%), sputum production (57.14%), throat discomfort (3.90%), hemoptysis (19.48%), chest pain (14.29%), dyspnea (61.04%), and respiratory failure (15.58%) (15). Atypical symptoms such as anorexia (1.30%) and dysphagia (1.30%) were also observed in some cases (1, 16). Physical examination may reveal rales and/or rhonchi upon pulmonary auscultation, as well as digital clubbing (7.79%), tachycardia, tachypnea, and respiratory failure.

At present, whether MKS is congenital or acquired is still under debate (17). Two siblings with MKS were reported in the literature, as well as possible MKS cases in Cousins, leading to the initial belief that MKS was an autosomal recessive disorder (5). It was later found that MKS was also seen in patients with Tetany, Ehlers-Danlos syndrome, Marfan syndrome, and cutis laxa (8).

In 2014, Mitterbauer et al. performed a chromosomal analysis and an array-CGH to search for genetic abnormalities. The MKS patient’s genome was completely unremarkable, the karyogram as well as the genome hybridization showed no significant gain or loss in known coding regions. Histological and immunohistochemical findings indicate that MKS is a chronic inflammatory condition characterized by the matrix metalloproteinase-mediated degradation of submucosal elastic fibers (18).

More and more people are recognizing that MKS is a secondary condition, associated with numerous chronic muscular, respiratory, or autoimmune diseases. These include pulmonary fibrosis, cystic fibrosis, bronchiectasis, emphysema, chronic bronchitis, amyotrophic lateral sclerosis (ALS) (19), sarcoidosis, chronic progressive histoplasmosis, giant cell arteritis (20), ankylosing spondylitis, rheumatoid arthritis, primary tracheobronchial amyloidosis (21), Sjogren’s syndrome (22), and Homocystinuria (23).

In addition, MKS has also been observed in a number of conditions found in critically ill patients requiring prolonged hyperinflation of their endotracheal or tracheostomy tube cuffs; including those with acute respiratory distress syndrome (ARDS) and respiratory failure (8). Tracheal enlargement is a recognized complication of prolonged tracheal intubation, especially when cuff pressure exceeds 30 cmH_2_O (24). In recent years, cases of MKS have also been reported in patients with COVID-19 (3, 25, 26).

Furthermore, Parris and Johnson described a case of MKS secondary to radiotherapy for the treatment of oropharyngeal carcinoma (26). Mohammed et al. described a case of fluoroquinolone-induced MKS, in which the diameter of the right main bronchus increased from 16.2 mm to 22.5 mm and the left main bronchus increased from 17.4 mm to 19.7 mm after 2 years of treatment (27).

Unfortunately, due to the limited understanding of the condition, there is scant evidence regarding effective remedies. And there are few interventions specifically targeting anatomical airway abnormalities, with treatment primarily focused on supportive symptomatic care. In our series of 77 cases, the treatment approaches were predominantly supportive care (67 cases), followed by tracheal stent placement (3 cases), tracheostomy tube placement (5 cases), lung transplantation (3 cases), and one case in which the specific treatment method was not mentioned.

In fact, for patients with both acute and chronic MKS, bronchodilators, mucolytic therapy, and intermittent steroid therapy have been shown to be beneficial. Appropriate antibiotic treatment is necessary for cases with bacterial infections. Additional supportive care options may include mobilization assistance and chest physical therapy to facilitate the clearance of secretions in these patients.

The management of chronic MKS in patients primarily focuses on symptom control and prevention of future infections. Firstly, smoking cessation is highly advantageous. Secondly, it is recommended to administer influenza and pneumococcal vaccinations to all adult MKS patients, irrespective of age or symptomatic status (5). Noninvasive mechanical ventilation (NIMV) has the potential to enhance expiratory flow and manage symptoms by reducing lung resistance and respiratory work load. In outpatient settings, some chronic patients have experienced symptom improvement with noninvasive continuous positive airway pressure (CPAP). However, it is important to note that NIMV may only provide temporary relief of clinical symptoms and may not achieve the intended treatment outcomes.

In patients with critical illness, if supportive care and medication fail to achieve desired outcomes, it may be necessary to consider surgical interventions. These interventions could encompass placement of a tracheostomy tube, tracheal stent placement, tracheobronchoplasty, laser therapy, or lung transplantation (15).

Due to the enlarged diameter of the airway, Ushakumari et al. recommend the use of endotracheal tubes with the largest diameter, through the glottic opening, an inflatable cuff to prevent air leakage, and wet gauze if necessary to reduce further leakage (7). Cheon et al. also chose a thicker tracheal tube than usual (inner diameter 8.0 mm, outer diameter 11.0 mm, maximum cuff size 28 mm) (28). Kim et al. observed that the standard tracheostomy tube is insufficient in length for proper placement within the dilated trachea of patients with MKS, resulting in significant peritubular sleeve leakage and subsequent hypercapnia. During tracheotomy and insertion, an undiagnosed case presented with tracheal enlargement secondary to massive cuff leakage resulting in ventilation failure (24). Hence, a customized tracheal catheter was shortened and carefully placed into the tracheostomy (3).

For patients with MKS, a primary anesthesia consideration is air leakage around the tracheal cannula, resulting in anesthetic gas leakage and inhalation hazards, catheter detachment, and passive airway collapse. These risks can complicate the process of obtaining adequate humidification during mechanical ventilation (28). Therefore, for MKS patients undergoing anesthesia, epidural anesthesia or lumbar anesthesia may be considered as alternative approaches (29).

Stent placement poses a significant challenge due to the need for using a large stent to stabilize an already enlarged airway, leading to common complications such as stent migration and mucus plug impaction. To mitigate the risk of stent migration, Y-type stents have been utilized with improved outcomes. Additionally, tracheobronchoplasty offers an alternative approach by restoring normal airway anatomy while preserving mucociliary function (12). However, stent implantation also has complications such as secretion retention, infection and restenosis due to granulation.

Laser bronchoplasty represents a novel and minimally invasive technique, capable of mitigating local nodule contraction and scar formation, thereby ameliorating airway collapse. Both domestic and international studies have reported favorable clinical outcomes, with patients experiencing significant improvement in symptoms within 1 week post-surgery. Consequently, laser bronchoplasty stands as a safe and effective approach for addressing cartilaginous nodules to achieve recanalization. Nonetheless, further research is warranted to comprehensively evaluate the efficacy of this technique (30).

Surgical intervention and lung transplantation are reserved for patients with advanced disease or those who have exhausted options such as CPAP, airway stenting, tracheoplasty, and laser therapy (23, 31). However, technical challenges arise due to the need to connect the graft to the original dilated bronchus. Surgical treatment carries significant risks and complications, with certain limitations impacting patient outcomes.

## Conclusion

Overall, there is a bias present in this study. The majority of cases are sourced from previously published journals, resulting in a focus on imaging descriptions and treatment modalities, and lack descriptions of patients’ symptoms and signs. In addition, two key conclusions can be drawn from our patients’ data: First, clinicians and radiologists have insufficient understanding of this disease, leading to frequent missed diagnosis, misdiagnosis, or delayed diagnosis. Although this syndrome is extremely rare, it significantly impacts patients’ quality of life. Diagnosis and management require a comprehensive assessment, including computed tomography and a multidisciplinary approach involving pulmonologists and radiologists. Understanding its clinical features, association with other respiratory diseases, and treatment options is critical to managing this rare respiratory disease. Second, the tracheal diameter of MKS has the potential to be reversible with appropriate treatment. This study reports the first documented case of reversible tracheal diameter in MKS patients.

## Data Availability

The original contributions presented in the study are included in the article/Supplementary material, further inquiries can be directed to the corresponding author.
