# Supplementary material for: Case Report: A case of reversible tracheal diameter Mounier-Kuhn syndrome and literature review
Source: Front Med (Lausanne). 2025 Apr 28;12:1544843. doi: 10.3389/fmed.2025.1544843 (PMC12067937; doi:10.3389/fmed.2025.1544843)
Supplement: Supplementary file 1 [file Supplementary_file_1.docx]

| **Table 2. Cases selected for inclusion** | | | | | | | |
| --- | --- | --- | --- | --- | --- | --- | --- |
| Authors, years | Age/Gender | Diamater Of Trachea（mm） | Right/Left Main Bronchus（mm） | MKS Type | Symptoms On Presentation | Final Diagnosis | Treatment |
| Mitterbauer, 2014[1] | 39/male | 36 | 17/20 | Type 2 | Dry cough | MKS, COPD | lung transplantation |
| Gupa,2014[2] | 70/male | 33 | 25/- | Type 1 | Dry cough, Respiratory failure | MKS | symptomatic and supportive treatment |
| Özdemir,2014[3] | 58/male | 35 |  | Type 1 | Productive cough， | MKS, hypertension, hypothyroiditis and type II diabetes | tracheal stent placement |
| Desiderio,2014[4] | 69/male | 57 | 38/32 | Type 1 | Fever, Productive cough | MKS, hypertension, hyperlipidaemia and a pneumothorax | symptomatic and supportive treatment |
| Marioara,2014[5] | 65/male | 59.1 | 30.4/20.2 | Type 3 | Productive cough, Chest pain, Weight loss | MKS, COPD | symptomatic and supportive treatment |
| Kumar,2014[6] | 34/male | 30 | 19.5/18.5 | Type 3 | Dry cough, Chest pain | MKS | symptomatic and supportive treatment |
| Lerner,2014[7] | 34/male | 44.2 |  | Type 1 | Fever, Productive cough, Hemoptysis | MKS | symptomatic and supportive treatment |
| Gouder,2014[8] | 43/male | 35 | 18/20 | Type 1 | Productive cough | MKS | symptomatic and supportive treatment |
| Singh,2015[9] | 54/male | 45.5 |  | Type 3 | Productive cough, Club-finger | MKS, asthma | symptomatic and supportive treatment |
| Uddin,2015[10] | 42/male | 40 |  | Type 1 | Pharyngeal discomfort | MKS, Primary tracheobronchial amyloidosis | symptomatic and supportive treatment |
| Lee,2015[11] | 51/male | 43.77 |  | Type 1 | Dyspnea | MKS, amyotrophic lateral sclerosis (ALS) | tracheal intubation |
| Sudhakar,2015[12] | 51/male | 40 | 42.4/25.8 | Type 1 |  | MKS, hypertension, depression and anxiety | lung transplantation |
| Schiettecatte,2015[13] | 38/male | 40.81 | 22.45/27.13 | Type 2 | Dry cough, Hemoptysis，Chest pain | MKS | symptomatic and supportive treatment |
| Unlu,2016[14] | 63/male | 29 | 23/20 | Type 1 | Dyspnea | MKS | symptomatic and supportive treatment |
| Govindaraj,2016[15] | 36/male | 35 | 25/24 | Type 1 | Productive cough | MKS | symptomatic and supportive treatment |
| Boglou,2016[16] | 83/female | 23 | 19.8/17.4 | Type 1 | Productive cough，Hemoptysis | MKS, IPF, left ovarian cancer, arterial hypertension, diabetes mellitus and osteoporosis | symptomatic and supportive treatment |
| Mondoni,2016[17] | 45/male | 60.1 | 24.2/23.1 | Type 3 | Productive cough，Dyspnea | MKS | symptomatic and supportive treatment |
| Cook,2016[18] | 44/male | 30 |  | Type 1 | Dry cough, Dyspnea, Respiratory failure | MKS | lung transplantation |
| Akgedik,2016[19] | 60/female | 36.5 |  | Type 3 | Dry cough，Hemoptysis，Dyspnea | MKS, asthma, pulmonary aspergillomas | symptomatic and supportive treatment |
| Casso,2016[20] | 84/male | 34.1 | 22.9/21.7 | Type 1 |  | MKS, aortic valve stenosis | tracheal intubation |
| Sullivan,2016[21] | 67/male | 32 |  | Type 2 | Dry cough,Dysphagia | MKS, mantle cell lymphoma |  |
| Fletcher,2017[22] | 45/female | 34 | 24/16 | Type 2 | Fever, Productive cough, Dyspnea, Respiratory failure | MKS, COPD, Human Immunodeficiency Virus | symptomatic and supportive treatment |
| Kaya,2017[23] | 60/male | 32 | 22/18 | Type 3 | Productive cough, Dyspnea, Respiratory failure, Club-finger | MKS, COPD | symptomatic and supportive treatment |
| Lee,2017[24] | 75/male | 46.32 |  | Type 1 | Productive cough | MKS | symptomatic and supportive treatment |
| Kuwal,2017[25] | 30/male | 31.7 | 19.6/20.5 | Type 3 | Fever, Productive cough | MKS, TB | symptomatic and supportive treatment |
| Chenbhanich,2017[26] | 79/male | 31 |  | Type 1 | Fever, Productive cough, Dyspnea，Respiratory failure | MKS, IPF | symptomatic and supportive treatment |
| Akgedik,2018[27] | 80/male | 34.3 | 22.8/21.2 | Type 1 | Dry cough, Dyspnea | MKS | symptomatic and supportive treatment |
|  | 41/male | 37 | 24.1/22.5 | Type 2 | Productive cough，Hemoptysis | MKS, TB | symptomatic and supportive treatment |
|  | 72/male | 32.3 | 22.3/22 | Type 2 | Dyspnea | MKS, IPF | symptomatic and supportive treatment |
|  | 38/male | 30.1 | 21.2/19 | Type 3 | Productive cough | MKS | symptomatic and supportive treatment |
|  | 67/male | 33 | 22.2/21 | Type 2 | Productive cough，Hemoptysis | MKS, COPD | symptomatic and supportive treatment |
|  | 59/male | 30.8 | 21/19.7 | Type 1 | Productive cough，Hemoptysis | MKS, COPD | symptomatic and supportive treatment |
|  | 63/male | 35 | 23.7/21.9 | Type 1 | Dry cough，Hemoptysis，Dyspnea | MKS, Pulmonary embolism, COPD | symptomatic and supportive treatment |
|  | 65/male | 28 | 20.7/19.5 | Type 2 | Dry cough，Dyspnea | MKS | symptomatic and supportive treatment |
|  | 74/male | 35.8 | 23/22 | Type 1 | Productive cough | MKS | symptomatic and supportive treatment |
|  | 63/male | 30.8 | 19.5/19 | Type 1 | Productive cough，Hemoptysis | MKS, TB | symptomatic and supportive treatment |
|  | 71/male | 37.5 | 24/23.2 | Type 1 | Dry cough，Dyspnea | MKS, COPD | symptomatic and supportive treatment |
| Geng,2018[28] | 79/male | 36 | 24/22 | Type 2 | Dyspnea | MKS, pulmonary fibrosis and Sjogren's syndrome | tracheal intubation |
| DAMIAN,2018[29] | 73/male | 37 | 22/25 | Type 2 | Dry cough, Dyspnea, Pharyngeal discomfort, Weight loss | MKS, Giant cell arteritis | symptomatic and supportive treatment |
| No,2019[30] | 82/male | 32 | 21.08/18.65 | Type 1 | Asymptomatic | MKS, falcine meningioma, atrial fibrillation and Parkinson's disease | symptomatic and supportive treatment |
| Punjadath,2019[31] | 21/male | 30.6 |  | Type 3 | Fever, Productive cough, Hemoptysis，Dyspnea, Chest pain, Respiratory failure, Club-finger | MKS | symptomatic and supportive treatment |
| Naciri,2019[32] | 81/male | 32 | 26/25 | Type 3 | Chest pain | MKS | symptomatic and supportive treatment |
| Santos,2019[33] | 49/male | 35 | 18/- | Type 3 | Respiratory failure | MKS | symptomatic and supportive treatment |
| Aguiar,2019[34] | 40/male | 28.5 |  | Type 3 | Fever, Productive cough | MKS | symptomatic and supportive treatment |
| Harper,2020[35] | 58/male | 52.2 |  | Type 1 | Respiratory failure | MKS | symptomatic and supportive treatment |
| Jafari,2020[36] | 51/male | 39 | 30/26 | Type 1 | Fever, dry cough，Dyspnea | MKS, COVID-19 | symptomatic and supportive treatment |
| Yazici,2020[37] | 25/male | 33.3 | 24/- | Type 1 | Dry cough，Productive cough，Hemoptysis | MKS | symptomatic and supportive treatment |
| Awad,2020[38] | 69/male |  | 22.5/19.7 | Type 1 | Hemoptysis，Dyspnea | MKS, COPD | symptomatic and supportive treatment |
| Estrada,2020[39] | 45/male | 35 |  | Type 3 | Dyspnea，Respiratory failure | MKS, severe pulmonary artery systolic pressure | symptomatic and supportive treatment |
| Satia,2020[40] | 49/male | 47.39 | 15/16 | Type 2 | Fever, Productive cough, Dyspnea | MKS, gastro-oesophageal reflux diseases (GORD) | symptomatic and supportive treatment |
| Fernández-Trujillo,2020[41] | 45/male | 33 | 22/17 | Type 3 | Productive cough，Dyspnea，Respiratory failure | MKS, arterial hypertension, iron deficiency anemia and auricular fibrillation | symptomatic and supportive treatment |
| Loued,2020[42] | 46/female | 42 | 22/24 | Type 2 | Productive cough，Chest pain | MKS | symptomatic and supportive treatment |
| Mkandawire,2020[43] | 43/female | 20.2 | 22.5/20.4 | Type 2 | Productive cough, Dyspnea, Chest pain | MKS, human immunodeficiency virus infection | symptomatic and supportive treatment |
| Wegner,2020[44] | 38/female | 33 | 25/29 | Type 3 | Productive cough | MKS | symptomatic and supportive treatment |
| Chandran,2020[45] | 41/male | 32.1 | 20/20 | Type 3 | Pharyngeal discomfort | MKS | symptomatic and supportive treatment |
| Shahin,2021[46] | 28/male | 31 | 21/24 | Type 3 | Productive cough, Dyspnea, Chest pain | MKS | symptomatic and supportive treatment |
| O'Bryan,2021[47] | 72/female | 32 |  | Type 1 | Fever, Productive cough, Dyspnea, Respiratory failure, Club-finger | MKS | symptomatic and supportive treatment |
| Rjimati,2021[48] | 67/male | 34.7 | 17/15 | Type 1 | Productive cough | MKS, Type 2 diabetes, hypothyroidism, gastroesophageal reflux disease | symptomatic and supportive treatment |
| Srivali,2021[49] | 65/male | 35 | 25/24 | Type 1 | Productive cough，Dyspnea | MKS | symptomatic and supportive treatment |
| Choudhury,2022[50] | 89/female | 29 | 15/14 | Type 1 | Dry cough, Productive cough，Dyspnea, Chest pain | MKS, COVID-19 | symptomatic and supportive treatment |
| Babirye,2022[51] | 43/male | 38.4 | 42.5/38.1 | Type 3 | Productive cough, Dyspnea, Chest pain, Weight loss, Club-finger | MKS, Pulmonary hypertension and right heart failure | symptomatic and supportive treatment |
| Imzil,2022[52] | 59/male | 47.3 | 23.4/35.5 | Type 2 | Productive cough，Hemoptysis，Dyspnea | MKS | symptomatic and supportive treatment |
| Suliman,2023[53] | 30/female | 26.87 |  | Type 1 | Productive cough，Dyspnea | MKS, homocystinuria | symptomatic and supportive treatment |
| Ayub,2023[54] | 62/male | 31.29 |  | Type 2 | Productive cough，Dyspnea，Respiratory failure, Club-finger | MKS | symptomatic and supportive treatment |
| Li,2023[55] | 69/male | 40.1 | 24.1/26.9 | Type 1 | Fever, dry cough，Productive cough，Dyspnea | MKS | symptomatic and supportive treatment |
| Li,2023[56] | 75/male | 34.2 | 24.2/24.8 | Type 2 | Productive cough，Dyspnea | MKS | symptomatic and supportive treatment |
| Wang,2023[57] | 64/male | 44.7 | 26.8/21.5 | Type 2 |  | MKS, Lung cancer | tracheal intubation |
| Zaghba,2023[58] | 67/female | 32 | 20/17.5 | Type 2 | Fever, Productive cough, Dyspnea | MKS | symptomatic and supportive treatment |
| Cheon,2024[59] | 94/female | 29.2 | 20.2/15.3 | Type 1 | Asymptomatic | MKS, emergency hemiarthroplasty of the hip under general anesthesia | tracheal intubation |
| Takimoto,2024[60] | 42/female | 26.9 |  | Type 3 | Dyspnea | MKS | symptomatic and supportive treatment |
| Ozgur,2024[61] | 78/male | 30.87 | 24.08/23.09 | Type 1 |  | MKS, COPD, met astatic squamous cell carcinoma primary to the lung | symptomatic and supportive treatment |
| Babiker,2024[62] | 69/male | 38 |  | Type 1 | Fever, dry cough, Hemoptysis, Dyspnea, Weight loss | MKS, COPD, pulmonary aspergillomas | symptomatic and supportive treatment |
| Chen,2024[63] | 34/male | 44.5 | 25.2/20.0 | Type 1 | Productive cough，Dyspnea | MKS | symptomatic and supportive treatment |
| Solís García,2024[64] | 46/female | 37 |  | Type 1 |  | MKS, asthma | symptomatic and supportive treatment |
| Li,2024[65] | 84/male | 62.6 | 30.9/37.9 | Type 1 | Fever, Productive cough | MKS, hyperlipemia, tracheobronchopathia osteochondroplastica (TO) | symptomatic and supportive treatment |
| Rossi,2024[66] | 40/male | 40 | 33/27 | Type 3 | Fever, Productive cough | MKS | symptomatic and supportive treatment |
| Our case,2024 | 82/male | 36.8 | 27/20.1 | Type 1 | Fever, Productive cough，Hemoptysis，Dyspnea, Anorexia | MKS, COPD, COVID-19 | symptomatic and supportive treatment |

**Reference：**

[1]KH Andreas Mitterbauer, Peter Birner, Michael Mildner, Helmut Prosch, Berthold Streubel, Shahrokh Taghavi, Walter Klepetko, Hendrik Jan Ankersmit (2014) Clinical‐radiological, histological and genetic analyses in a lung transplant recipient with Mounier–Kuhn syndrome and end‐stage chronic obstructive pulmonary disease.The Clinical Respiratory Journal 9(3):375-9.doi:10.1111/crj.12139

[2]UG P Gupa, A Bhalla, et al. (2014) Mounier-Kuhn syndrome masquerading pulmonary thromboembolism in an elderly male.Lung India 31(1):10.4103/0970-2113.125995

[3]SS C Özdemir, L Karasulu, S Altın, L Dalar (2014) Tracheomalacia Treatment Using a Large-Diameter, Custom-Made Airway Stent in a Case with Mounier-Kuhn Syndrome.Case Reports in Pulmonology 2014(1-4.doi:10.1155/2014/910135

[4]JF Michael Desiderio, Vijaiganesh Nagarajan (2014) Recurrent pneumonia as a result of Mounier–Kuhn syndrome.

[5]VP S Marioara, A Fabian (2014) Mounier-kuhn Syndrome.Bronchoscopic Image 21(145-9.doi:

[6]AKM Saurabh Kumar (2014) Mounier-Kuhn syndrome (MKS) - Pathognomonic Findings.Journal of Clinical and Diagnostic Research 8(12):RJ01-RJ2.doi:10.7860/jcdr/2014/10829.5325

[7]R Charles Lerner, KJ Patel (2014) The Man with the Large Trachea: Mounier-Kuhn Syndrome.The American Journal of Medicine 127(11):1072-4.doi:10.1016/j.amjmed.2014.06.033

[8]DB Caroline Gouder, Peter Fsadni, Stephen Montefort (2014) A delayed diagnosis of Mounier-Kuhn syndrome.BMJ Case Report 2014(bcr2014203674.doi:10.1136/bcr-2014-203674

[9]RK Mandeep Singh (2015) A 54-year-old Man with Tracheomegaly, Tracheal Diverticulas and Bronchiectasis- Mounier-Kuhn Syndrome.Indian J Chest Dis Allied Sci 57(113-5.doi:

[10]DR A. K. M. Nizam Uddin, MWF Mansfield, Kenneth K. Lau (2015) Primary tracheobronchial amyloidosis associated with tracheobronchomegaly evaluated by novel four‐dimensional functional CT.Respirology Case Reports 3(4):151-4.doi:10.1002/rcr2.134

[11]DH Lee, TM Yoon, JK Lee, SC Lim (2015) Tracheomegaly Secondary to Tracheotomy Tube Cuff in Amyotrophic Lateral Sclerosis.Medicine 94(42):10.1097/md.0000000000001763

[12]S Subramani, B Freeman, S Rajagopal (2015) Anesthetic Considerations for Bilateral Lung Transplantation in Mounier-Kuhn Syndrome.Journal of Cardiothoracic and Vascular Anesthesia 29(3):727-30.doi:10.1053/j.jvca.2013.10.030

[13]EVH E.Schiettecatte, R. Van Herreweghe, K. Verstraete (2015) Mounier-Kuhn Syndrome.JBR-TBR 98(139-40.doi:

[14]EN Unlu, AN Annakkaya, EG Balbay, LY Aydın, S Safcı, M Boran, et al. (2016) An unusual cause of recurrent spontaneous pneumothorax: the Mounier-Kuhn syndrome.The American Journal of Emergency Medicine 34(1):122.e1-.e2.doi:10.1016/j.ajem.2015.05.050

[15]V Govindaraj, M Mohanty Mohapatra, B Nagamalli Kumar, S Narayanasami (2016) Tracheobronchomegaly as a Cause of Bronchiectasis in an Adult.Case Reports in Pulmonology 2016(1-4.doi:10.1155/2016/5049406

[16]P Boglou, N Papanas, A Oikonomou, S Bakali, P Steiropoulos (2016) Mounier-Kuhn Syndrome in an Elderly Female with Pulmonary Fibrosis.Case Reports in Medicine 2016(1-4.doi:10.1155/2016/8708251

[17]M Mondoni, P Carlucci, E Parazzini, P Busatto, S Centanni (2016) Huge Tracheal Diverticulum in a Patient with Mounier-Kuhn Syndrome.European Journal of Case Reports in Internal Medicine 3(5):10.12890/2016_000419

[18]DP Cook, RJ Adam, MH Abou Alaiwa, M Eberlein, JA Klesney‐Tait, KR Parekh, et al. (2016) Mounier–Kuhn syndrome: a case of tracheal smooth muscle remodeling.Clinical Case Reports 5(2):93-6.doi:10.1002/ccr3.794

[19]R Akgedik, C Eren Dagli, AB Kurt, H Ozturk, N Tas (2016) The Association of Mounier-Kuhn Syndrome and Pulmonary Aspergillomas: A Case Report.Balkan Medical Journal 33(5):585-6.doi:10.5152/balkanmedj.2016.150397

[20]G Casso, P Schoettker (2016) Tracheobronchomegaly.New England Journal of Medicine 374(12):10.1056/NEJMicm1508462

[21]S Sullivan (2016) An Unusual Cause of Dysphagia.Dysphagia 31(5):717-8.doi:10.1007/s00455-016-9706-x

[22]A Fletcher, J Stowell, S Jamoulis (2017) Congenital Tracheobronchomegaly (Mounier-Kuhn Syndrome) in a Woman with Human Immunodeficiency Virus: A Case Report.Cureus 10.7759/cureus.1136

[23]AG Kaya, A Çiledağ, Ç Atasoy, D Karnak (2017) Flexible bronchoscopy and mechanical ventilation in managing Mounier-Kuhn syndrome: a case report.Sao Paulo Medical Journal 136(3):266-9.doi:10.1590/1516-3180.20160336270117

[24]C-C Chuang, C-C Lee, B-S Lin, J-Y Chen (2017) Anesthesia for a patient with unexpected giant tracheobronchomegaly.Tzu Chi Medical Journal 29(1):10.4103/tcmj.tcmj_1_17

[25]A Kuwal, N Dutt, N Chauhan, S Kumar, S Purohit, LK Saini (2017) An Atypical Case of Mounier-Kuhn Syndrome.Journal of Bronchology & Interventional Pulmonology 24(1):84-7.doi:10.1097/lbr.0000000000000226

[26]J Chenbhanich, J Villa-Camacho, J Konter (2017) A case of tracheobronchomegaly.European Journal of Internal Medicine 42(e7-e8.doi:10.1016/j.ejim.2017.01.011

[27]R Akgedik, H Karamanli, D Kizilirmak, AB Kurt, H Öztürk, BB Yildirim, et al. (2018) Mounier‐Kuhn syndrome (tracheobronchomegaly): An analysis of eleven cases.The Clinical Respiratory Journal 12(3):885-9.doi:10.1111/crj.12600

[28]Y Geng, J Zhou, Z Liu (2018) Tracheobronchomegaly in Intubated Ventilation of ARDS.Archivos de Bronconeumología 54(2):10.1016/j.arbres.2017.05.019

[29]SM LO Damian, CAM Pamfil, L Rogojan, S Rednic, AA Maniu, M Poenaru (2018) Tracheal enlargement or Mounier-Kuhn syndrome in giant cell arteritis: a possible causal association with therapeutic implications.Romanian Journal of Morphology & Embryology 59(2):595-9.doi:

[30]H-J No, J-M Lee, D Won, P Kang, S Choi (2019) Airway management of a patient incidentally diagnosed with Mounier-Kuhn syndrome during general anesthesia.Journal of Dental Anesthesia and Pain Medicine 19(5):10.17245/jdapm.2019.19.5.301

[31]S Punjadath, K Madan, A Mohan, S Mittal (2019) A young man with chronic cough: big is not always beautiful.Thorax 74(10):1006-7.doi:10.1136/thoraxjnl-2019-213360

[32]S Naciri, R Zahraoui, M Soualhi, J-E Bourkadi (2019) An Unusual Cause of Spontaneous Pneumomediastinum: The Mounier-Kuhn Syndrome.Case Reports in Pulmonology 2019(1-5.doi:10.1155/2019/5359309

[33]TKT Rômulo Florêncio Tristão Santos, Isa Félix Adôrno, Edson Marchiori, Thiago Franchi Nunes (2019) Mounier-Kuhn syndrome: an unusual cause of bronchiectasis.Radiologia Brasileira 52(2):130-1.doi:10.1590/0100-3984.2017.0167

[34]F Aguiar, C Pacheco, P Silveira (2019) Mounier-Kuhn Syndrome.Archivos de Bronconeumología (English Edition) 55(12):10.1016/j.arbr.2019.01.026

[35]S Harper, M Robinson, G Manning, A Jones, J Hobson, CL Shelton (2020) Management of tracheostomy‐related tracheomegaly in a patient with COVID‐19 pneumonitis.Anaesthesia Reports 8(2):159-62.doi:10.1002/anr3.12076

[36]R Jafari, L Cegolon, F Dehghanpoor, M Javanbakht, M Izadi, SH Saadat, et al. (2020) Early manifestation of ARDS in COVID-19 infection in a 51- year-old man affected by Mounier-Kuhn syndrome.Heart & Lung 49(6):855-7.doi:10.1016/j.hrtlng.2020.05.005

[37]O Yazici, S Gulen, E Ceylan (2020) The Effect of Ankaferd Blood Stopper Used for Massive Hemoptysis in a Patient With Mounier-Kuhn Syndrome: A Case Report.Journal of the Pakistan Medical Association 0):10.5455/jpma.295533

[38]SI Mohammed T. Awad, Samantha L. Spetz,Ahmad Kattan, Momen Banifadel, Kristopher Arndt, Taha Sheikh, Ragheb Assaly (2020) Tracheobronchial Dilation (Mounier–Kuhn-like Syndrome) Secondary to Fluoroquinolones.American Journal of Therapeutics

[39]DE Estrada, FL Uribe-Buritica, CA Vargas, C García, W Martínez (2020) Recurrent Pneumonias in a Previously Healthy and Immunocompetent Young Adult: A Case Report Mounier-Kuhn Syndrome.American Journal of Case Reports 21(10.12659/ajcr.918535

[40]I Satia, B Dua, N Singh, K Killian, PM O'Byrne (2020) Tracheobronchomegaly, cough and recurrent chest infection: Mounier-Kuhn syndrome.ERJ Open Research 6(2):10.1183/23120541.00138-2020

[41]L Fernández-Trujillo, S Sangiovanni, EI Morales, LF Sua, CA García (2020) Thoracic Computed Tomography Scan and Bronchoscopy Appearance of Mounier-Kuhn Syndrome: A Case Report.Journal of Investigative Medicine High Impact Case Reports 8(10.1177/2324709620947892

[42]L Loued, A Migaou, A Achour, A Ben Saad, SC Mhammed, N Fahem, et al. (2020) Mounier-Kuhn syndrome: A variable course disease.Respiratory Medicine Case Reports 31(10.1016/j.rmcr.2020.101238

[43]NMM Mercy Juliette Mkandawire, Ngalawi Mraba (2020) A curious case of cough: Mounier-Kuhn yndrome in a Namibian female patient.

[44]F Wegner, J Barkhausen (2020) CT of Mounier-Kuhn Disease.Radiology 294(2):246-.doi:10.1148/radiol.2019191791

[45]A Chandran, P Sagar, AS Bhalla, R Kumar (2020) Mounier-Kuhn syndrome.BMJ Case Reports 14(1):10.1136/bcr-2020-239876

[46]HT Shahin S, Van Es W. Grutters J, Mateyo K (2021) Congenital tracheobronchomegaly (Mounier-Kuhn syndrome) in a 28-year-old Zambian male: a case report.Pan African Medical Journal 40(153):10.11604/pamj.2021.40.153.31703

[47]C J.O'Bryan, Ronald Espinosa, Subramanyam Chittivelu, V Wrenn (2021) Recurrent Lower Respiratory Tract Infections Due to Mounier-Kuhn Syndrome.Cureus 13(6):e15437.doi:10.7759/cureus.15437

[48]M Rjimati, M Serraj, M Elbiaze, MC Benjelloun, B Amara (2021) Mounier-Kuhn syndrome (Tracheobronchomegaly): Radiological diagnosis.Radiology Case Reports 16(9):2546-50.doi:10.1016/j.radcr.2021.06.021

[49]N Srivali, F De Giacomi (2021) Mounier-Kuhn Syndrome: A Rare Cause of Recurrent Chest Infection.Archivos de Bronconeumología (English Edition) 57(10):10.1016/j.arbr.2020.12.011

[50]S Choudhury, A Chohan, PT Taweesedt, R Dadhwal, A Vakil (2022) Coronavirus Disease 2019-Induced Tracheomegaly: A Case Report.Cureus 14(4):e23810.doi:10.7759/cureus.23810

[51]D Babirye, J Walubembe, JA Babirye, JB Baluku, P Byakika-Kibwika, E Nabawanuka (2022) Tracheobronchomegaly (Mounier-Kuhn Syndrome) in a 43-Year-Old Male: A Case Report.International Medical Case Reports Journal Volume 15(631-7.doi:10.2147/imcrj.S386083

[52]A Imzil, F Bounoua, HN Amrani, H Moubachir, H Serhane (2022) Tracheobronchomegaly (Mounier-Kuhn Syndrome) with CT and bronchoscopic correlation: A case report.Radiology Case Reports 17(10):3611-5.doi:10.1016/j.radcr.2022.06.077

[53]AM Suliman, MA Alamin, MM Hamza (2023) Tracheobronchomegaly (Mounier-Kuhn syndrome) and Bronchiectasis as rare manifestations of Homocystinuria.Respiratory Medicine Case Reports 42(10.1016/j.rmcr.2023.101808

[54]II Ayub, K Vengadakrishnan (2023) Mounier-Kuhn Syndrome.Tuberculosis and Respiratory Diseases 86(1):59-60.doi:10.4046/trd.2022.0123

[55]S Li, L Dong, L Zhi (2023) Tracheobronchomegaly: A rare but easily misdiagnosed disease.Asian Journal of Surgery 46(11):5314-5.doi:10.1016/j.asjsur.2023.07.073

[56]ZH Li, RJ Wang, S Gao (2023) Tracheobronchomegaly (Mounier-Kuhn syndrome): a case report.QJM: An International Journal of Medicine 116(9):792-3.doi:10.1093/qjmed/hcad109

[57]S-N Wang, A-S Wu, J-B Miao, S Chen, J Jiang (2023) Airway management for a patient with tracheobronchomegaly undergoing lobectomy: a case report.BMC Anesthesiology 23(1):10.1186/s12871-023-02324-5

[58]N Zaghba, Z Laklaai, K Chaanoun, H Benjelloun, N Yassine (2023) Mounier-Kuhn syndrome: a typical case including CT and bronchoscopic imaging.Oxford Medical Case Reports 2023(11):10.1093/omcr/omad123

[59]B Cheon, JH Lee, JH Kim, SM Hwang (2024) Airway management of a patient with Mounier-Kuhn syndrome during general anesthesia - A case report.Anesthesia and Pain Medicine 19(2):156-60.doi:10.17085/apm.23172

[60]T Takimoto, H Sumikawa, Y Inoue, T Arai (2024) Autofluorescence Imaging of Bronchoscopy in Mounier-Kuhn Syndrome: Negative Autofluorescence Sign.American Journal of Respiratory and Critical Care Medicine 210(5):665-8.doi:10.1164/rccm.202312-2233IM

[61]SS Ozgur, A Aiken, L Bathobakae, N Ansari, P Michael, M Ismail (2024) Incidental finding or the incognito culprit? A case of Mounier‐Kuhn syndrome.Clinical Case Reports 12(8):10.1002/ccr3.9259

[62]S Babiker, M Hajalamin (2024) An atypical encounter: Mounir-Kuhn syndrome and aspergilloma coexistence: A case report.Radiology Case Reports 19(9):3962-5.doi:10.1016/j.radcr.2024.06.001

[63]S-M Chen, G-W Chen (2024) A case description of tracheobronchomegaly combined with bronchiectasis and infection.Quantitative Imaging in Medicine and Surgery 14(6):4276-80.doi:10.21037/qims-24-41

[64]M Solís García, C Cisneros Serrano, AS Martín Hernández, JM Eiros Bachiller, C Marcos (2024) Mounier-Kuhn syndrome in poorly controlled asthma.Journal of Asthma 61(10):1351-4.doi:10.1080/02770903.2024.2344168

[65]ZH Li, L-X Kong, S Zhu, Y Hu, S Gao (2024) Tracheobronchomegaly associated with tracheobronchopathia osteochondroplastica: a case report.Frontiers in Medicine 11(10.3389/fmed.2024.1444995

[66]S Rossi, F Volpi, R Castellana, R Pancani, F Dente, R Gaeta, et al. (2024) A case report of unusual recurrent bronchopneumonia infections in Mounier-Kuhn syndrome.Radiology Case Reports 19(6):2525-30.doi:10.1016/j.radcr.2024.03.028
